# Dermatology Resident Billing and the Impact of 2021 Evaluation and Management Coding Changes

**DOI:** 10.7759/cureus.15810

**Published:** 2021-06-21

**Authors:** Michael Tassavor, Aatman Shah, Jonathan Ungar

**Affiliations:** 1 Dermatology, Icahn School of Medicine at Mount Sinai, New York, USA

**Keywords:** billing, coding, residency, revenue, residents, e/m, cpt

## Abstract

New billing rules were implemented in 2021 for determining the level of service in outpatient encounters. The purpose of this study was to assess overall dermatology resident billing at our institution and the impact of these rule changes. Billing codes from four months of our resident clinic were extracted from our electronic medical records (EMR) and analyzed. Nationwide Medicare data for dermatologists were used as a comparison. The coding changes were associated with a 13% increase in level 4 codes and a 20% decrease in level 2 codes. Overall, level 3 codes remained the most common codes submitted. Billing patterns were not concordant with nationwide Medicare utilization.

## Introduction

Academic medical centers depend heavily on resident physicians, particularly in outpatient clinics that largely see Medicaid populations. The financial viability of these clinics thus often rests on the billing performance of these residents. A recent survey of dermatology residents nationwide showed that while 79% of dermatology residents had formal billing instruction, only 37% felt confident in their knowledge [1]. Two papers examining resident billing accuracy of internal and family medicine residents each found annual losses of nearly $500,000 a year due to undercoding [2,3].

While attending physicians are nominally responsible for billing codes, practically, it is the resident who often determines them. Therefore, accurate billing knowledge in residents is important to both the residents and the institution employing them.

Current Procedural Terminology (CPT) codes, licensed from the American Medical Association (AMA), are the standard for billing in the United States. CPT codes are five-digit codes that encompass any service that can be billed for in healthcare. They are divided into various categories, including evaluation and management (E/M), anesthesia, surgery, radiology, pathology and laboratory, and medicine. Of these, E/M and surgery constitute the bulk of codes used in outpatient practice, representing Medical Decision Making (MDM) and procedures, respectively. E/M codes, which are broken down by levels of care (and thus reimbursement), have been the subject of significant review in the past decade.

E/M codes used to be determined in part by the amount of documentation in the history, review of systems, and physical exam. This was thought to contribute to significant amounts of “note bloat” and extra work for clinicians. Many other rules and exceptions made this system ripe for billing mistakes, resulting in costly audits, legal consequences, and unreimbursed work. Studies have shown a high level of variability among coding experts for the same documentation, which made simplifying E/M determination vitally important [4].

To address this problem, new rules were put in place for 2021, drastically changing the criteria for determining E/M codes [5]. MDM became the sole determining criteria, cutting most of the rules pertaining to the history and physical exam. While billing by time is also an option, it does not take into account resident time spent. 

To assess the impact of these changes, we analyzed billing data for dermatology residents in our residency program before and after the new billing rules were implemented. 

## Materials and methods

The study was conducted at a large, urban academic dermatology residency program. Billing data was queried from the electronic medical records (EMR) at our institution for a single resident clinic open three half days a week. This clinic sees exclusively state Medicaid patients and does not include dermatologic surgery, which is referred to a separate resident clinic. A two-month period before the billing changes was chosen - August and September 2020. A two-month period after the billing changes was chosen - January and February 2021. These months were non-contiguous due to the intervening months being downbooked due to coronavirus limitations. 

CPT codes corresponding to the E/M level of service were pulled for this time period and categorized by month and level of service. No personal identifiers - resident or patient - were gathered and all data was extracted in aggregate. No interventions were made in terms of billing education other than informing residents of the new rules. Unfortunately, statewide Medicaid utilization data was not readily available for comparison. Instead, 2018 Medicare utilization data for dermatologists - the latest available - was extracted from the publicly available CMS database [6]. 

## Results

There were an average of 687 unique patient visits per month in this specific clinic over the four studied months. This is not a reflection of all dermatology resident patient encounters as our institution has multiple dermatology resident clinics at different sites. 

**Figure 1:**
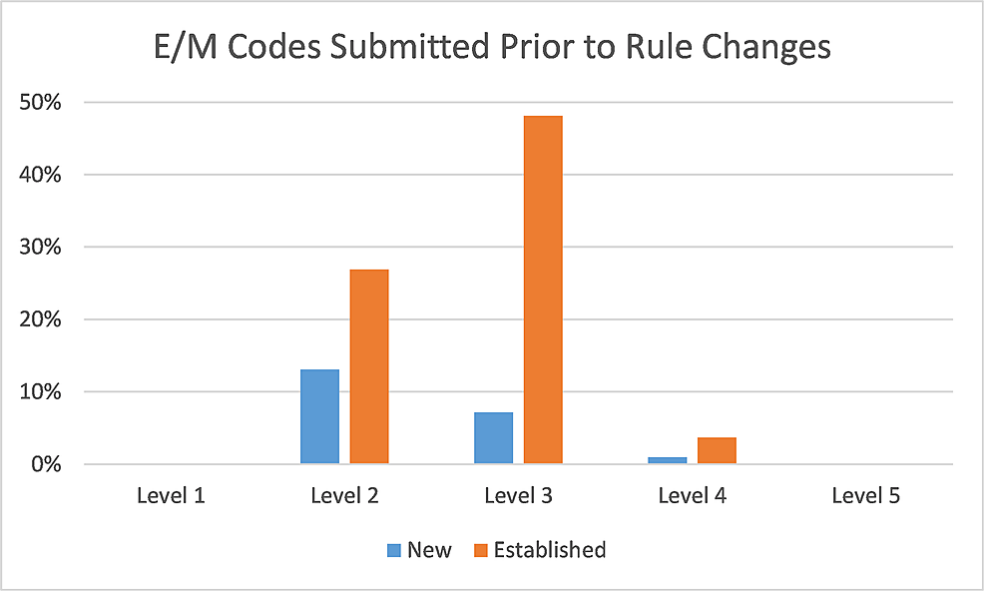
E/M Codes Submitted Prior to Rule Changes Evaluation and Management (E/M) billing codes submitted by residents (August through September 2020). Percentages in the Y axis reflect the percentage of all submitted E/M codes.

**Figure 2:**
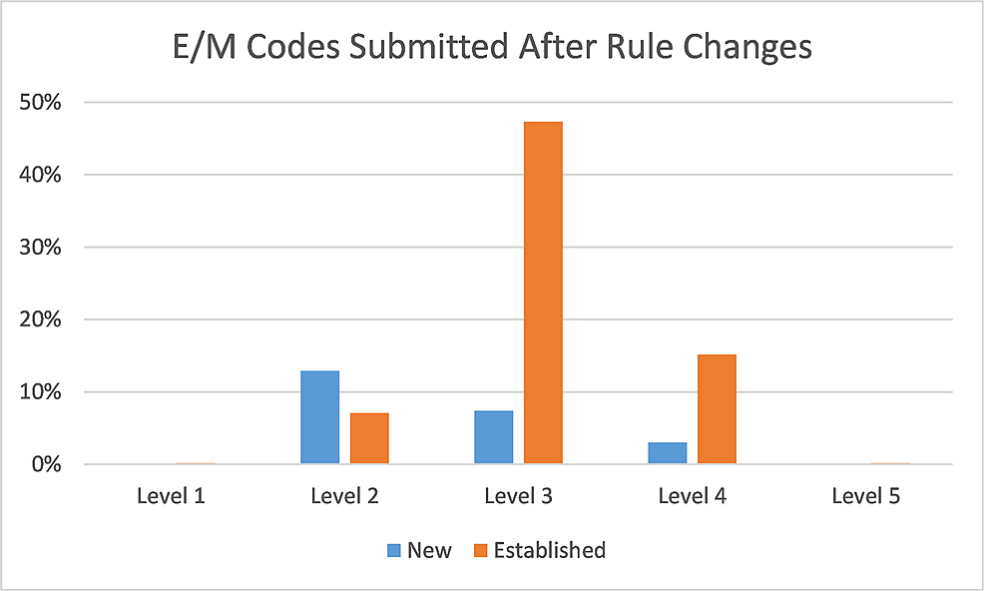
E/M Codes Submitted After Rule Changes Evaluation and Management (E/M) billing codes submitted by residents (January through February 2021). Percentages in the Y axis reflect the percentage of all submitted E/M codes.

Before and after the coding change, the vast majority of billed encounters were billed as a level 3. New patients were mostly billed as 99202, while established patients were mostly billed as 99213. There were minimal level 1 or level 5 codes submitted. The only notable change before and after the new billing rules was a 13% increase in level 4 codes and a 20% decrease in level 2 codes for both new and established patients. (Figures 1, 2)

**Figure 3:**
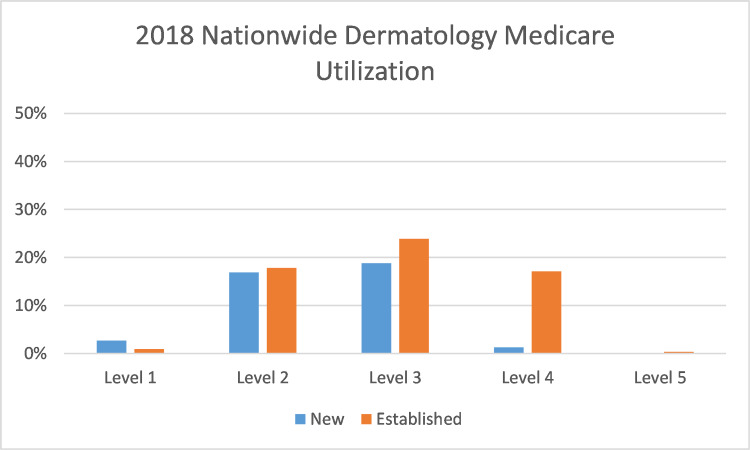
2018 Nationwide Dermatology Medicare Utilization Evaluation and Management (E/M) billing codes submitted by dermatologists nationwide to Medicare in 2018. Percentages in the Y axis reflect the percentage of all submitted E/M codes.

While level 3 codes were also the most common codes submitted to Medicare by dermatologists in 2018, the distribution between the codes was more even. (Figure 3)

## Discussion

To our knowledge, this is the first study detailing dermatology resident billing practices. Our findings add to previous studies involving resident billing in outpatient family medicine, internal medicine, and orthopedic surgery [2,3,7]. 

We found that the new billing rules yielded a modest increase in level 4 codes and a substantial decrease in level 2 codes, possibly due to the changes making it easier to bill at a higher level. Resident billing both before and after the new rules were far different than nationwide Medicare utilization. This could be explained by the different patient populations and disease complexities seen by academic medical centers compared to typical practices. 

This data and similar analysis at other residency programs can be used to monitor resident billing performance and reduce the incidence of underbilling. Residents billing far outside the norm can be identified and their billing knowledge remedied if necessary. After this study, our residency program has embarked on an ongoing prospective study determining the impact of an educational intervention on overall resident billing performance as determined by randomized chart reviews. 

Limitations to this study include the data being pulled from only a single institution over a limited time period. It is possible that giving the residents more time to acclimate to the new rules would have yielded greater changes. Moreover, these overall numbers may be altered by the ongoing COVID-19 pandemic, though the months chosen had few limitations in patient volume. The comparison to 2018 Medicare data is also not ideal given differences in geography, year, and likely patient populations, as noted above. Future work in this area could include the addition of procedural codes to better illustrate overall resident billing, though these would not be expected to change with the new E/M rules. 

## Conclusions

The 2021 E/M coding changes were associated with a modest increase in level 4 codes and a substantial decrease in level 2 codes in our dermatology residency program. This may be due to the intended goal of reducing coding complexity. Overall, level 3 codes remained by far the most common codes submitted. These trends were not concordant with nationwide Medicare data.
